# L-Carnitine enhances porcine sperm quality, longevity, and zona pellucida binding in cooled semen

**DOI:** 10.1590/1984-3143-AR2023-0143

**Published:** 2025-02-24

**Authors:** Monique de Albuquerque Lagares, Nathalia Abreu Amaral, Joyce Junia Braga, Natalia de Castro Alves, Marina Morra Freitas, Rafael Romero Nicolino, Raphael Rocha Wenceslau, Fernanda da Rocha Anselmo, Marina Maria do Carmo Silva Oliveira, Eduardo Damasceno Costa, Fernanda Radicchi Campos Lobato de Almeida, Rubens Stahlberg

**Affiliations:** 1 Escola de Veterinária, Universidade Federal de Minas Gerais, Belo Horizonte, MG, Brasil; 2 Instituto de Ciências Biológicas, Universidade Federal de Minas Gerais, Belo Horizonte, MG, Brasil; 3 Departamento de Medicina Veterinária, Pontifícia Universidade Católica, Betim, MG, Brasil

**Keywords:** spermatozoa, swine, boar, antioxidant, artificial insemination

## Abstract

Porcine breeding industries typically ensure the viability of boar artificial insemination doses during a 5-day liquid storage period at 17 °C. This study aimed to investigate whether the addition of L-carnitine (LC) to boar semen doses on different days of cooled storage could extend their usability. In experiment 1, LC was added to porcine semen doses on the fifth day (d5) of cooled storage performing five treatments control (no LC), 0.5, 1-, 5- and 10-mM LC. On d6 and d8 of storage, semen samples were evaluated for sperm motility and kinematic parameters, membrane functionality, and hydrogen peroxide and nitrite concentrations. In experiment 2, the number of sperm bound to the zona pellucida (ZP) was determined, as a way to investigate sperm penetration capability from boar insemination doses, with co-incubation with porcine oocytes. LC concentration that produced the most favorable outcomes in Experiment 1 was chosen to experiments 2 and 3, performing two treatments in the absence and with the LC. In Experiment 3, LC was added to cooled porcine semen doses after one day of storage (d1), and the same evaluations of experiment 1 were conducted on days 5, 7, 9, and 12, including sperm membrane integrity. The addition of 10 mM LC on d5 and d1 of storage improved sperm motility, which was extended up to 8 and 12 days of cooled storage, respectively. LC addition on d5 of storage increased sperm membrane functionality, while when added to semen on d1 of storage, it decreased NO_2_^-^ concentration on d9. On d6 of cooled storage 10 mM LC increased the number of sperm bound to ZP compared to the control. In conclusion, adding 10 mM LC to porcine semen doses at 17 °C improved sperm characteristics and ZP binding, ultimately enhancing sperm viability for up to 12d.

## Introduction

In porcine breeding, artificial insemination (AI) doses are typically cooled to 17 °C to decrease sperm metabolism and extend their longevity (Lopez Rodriguez et al., 2017; Szymanowicz et al., 2019; Waberski et al., 2019; Hensel et al., 2020; Flowers, 2022; Henning et al., 2022; Betancur et al., 2023; Restrepo et al., 2023). However, factors such as the cooling process, storage duration, and semen handling techniques contribute to increase the *in vitro* reactive oxygen species (ROS) production in semen (Bansal and Bilaspuri, 2010; Bandeira et al., 2016). ROS are molecules with the ability to exist independently, characterized by containing at least one oxygen atom and one or more unpaired electrons. This category encompasses oxygen free radicals, such as the superoxide anion radical, hydroxyl radical, hydroperoxyl radical, singlet oxygen, as well as free nitrogen radicals. Within physiological conditions, cells produce small amounts of ROS during various cellular processes, including aerobic respiration and inflammatory responses. Primarily serving as signaling molecules, ROS also play roles in inducing cell differentiation and apoptosis, thereby contributing to the natural aging process (Li et al., 2016). When it exceeds the sperm's antioxidant capacity, sperm viability is compromised (Bansal and Bilaspuri, 2010). ROS primarily target the sperm membrane (Ahmadi et al., 2016), which is particularly sensitive to oxidation in porcine sperm due to their high content of polyunsaturated fatty acids and limited antioxidant defenses (Lima and Abdalla, 2001; Nordberg and Arnér, 2001; Nogueira et al., 2014). The detrimental effects of ROS and nitrogen reactive species (RNS) on sperm include membrane disorganization, increased permeability, DNA damage, apoptosis induction, interference with mitochondrial energy generation, reduced motility, and impaired fertilization (Bansal and Bilaspuri, 2010; Bandeira et al., 2016). To mitigate these damages, antioxidants have been added to semen (Severo et al., 2011).

The addition of antioxidants to cooled semen could be a valuable practice for the suine industry, ensuring a high-quality cooled semen after five day of storage. This could be especially crucial for boars with sperm that do not tolerate longer storage periods. L-carnitine (LC) is an antioxidant amino acid, a highly polar, water-soluble quaternary amine found in tissues with high metabolic activity and reproductive systems, epididymal plasma, and sperm (Surai, 2015; Abd-Elrazek and Ahmed-Farid, 2018).

The antioxidant properties of LC are attributed to its ability to reduce substrate availability for lipid peroxidation (Stradaioli et al., 2004) by transporting fatty acids into the sperm mitochondrion for beta-oxidation, thereby generating ATP (Sariozkan et al., 2014). L-carnitine increases sperm motility by changing in fatty acid metabolism (Elokil et al., 2019). Additionally, LC stimulates the enzymatic antioxidant system (Tabatabaei and Aghaei, 2012; Gibb and Aitken, 2016), enhancing the activity of superoxide dismutase and glutathione peroxidase (Lisboa et al., 2014), and exhibits free-radical scavenging, anti-cytokine, and anti-apoptotic activities (Agarwal and Saleh, 2002; Gülçin, 2006). LC also regulates the flow of acetyl groups and energy balance through the cell membrane by facilitating the transport of free fatty acids and acetyl-CoA byproducts into the mitochondrion. The acetyl groups are temporarily associated with LC, producing L-acetyl-carnitine. Consequently, carnitine facilitates the transport of acetyl groups via L-acetyl-carnitine (Bremer, 1983; Gülçin, 2006). After absorption into the blood plasma, LC passively diffuses into the epididymis (Abd-Elrazek and Ahmed-Farid, 2018) and facilitates the transport of substrates across the mitochondrial cell membrane for energy production (Jeulin and Lewin, 1996).

The addition of LC to cooled semen has been reported in studies involving equines (Lisboa et al., 2014; Gibb et al., 2015; Gibb and Aitken, 2016), roosters (Partyka et al., 2017), and in the context of fresh porcine semen, albeit in high concentrations (Yang et al., 2020).

Further, incorporating analyses of gamete interactions, alongside sperm quality assessments, could offer an improved method for predicting male fertility (Gadea, 2005). The attachment and penetration of the zona pellucida (ZP) represent crucial obstacles that sperm must overcome in the fertilization process (Harrison, 1997; Larsson and Rodriguez-Martinez, 2000). A positive correlation between zona-binding ability and fertility has been established when estimated by average litter size (Braundmeier et al., 2004). Therefore, it is worth verifying whether the addition of low LC concentrations to cooled porcine semen has a beneficial effect on sperm characteristics, antioxidant activity, and zona pellucida binding capability. This procedure could be a valuable practice for the swine industry, particularly for boars whose sperm do not tolerate extended storage periods, ensuring high-quality cooled semen for a longer duration.

This study aimed to investigate whether the addition of LC to porcine cooled semen stored at 17 °C on different days prolongs sperm motility and kinematics, membrane integrity and functionality, concentrations of nitrite and hydrogen peroxide, and enhances binding to the ZP.

## Methods

All experimental procedures were performed according to the Brazilian ethical and animal welfare principles for the utilization and care of animals used in research and were approved by the ethical Committee “Comissão de ética no uso de animais” (Protocol number 40/2018) at the Faculty of Veterinary Medicine, Pontifical Catholic University (PUC-Minas) of Minas Gerais, Betim, Brazil.

All reagents were purchased from Sigma- Aldrich, Inc, St. Louis, MO unless otherwise stated.

### Experiment 1: Addition of LC to semen on day 5 of cooled storage

**Figure 1:**
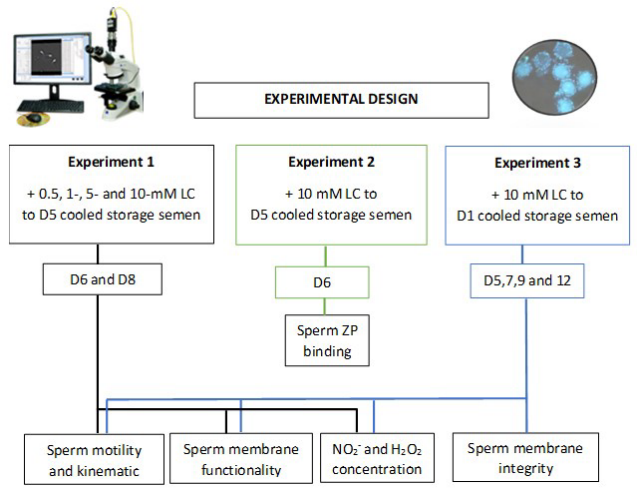
Schematic representation of the experimental design.

A schematic representation of the experimental design was presented in Figure 1. On d5 of storage at 17 °C, 10 porcine insemination doses (containing 100 mL each) were distributed into five separate 10 mL samples. These samples were then distributed into five experimental groups: control (no LC), and LC additions from different stock solutions (25, 50, 250, and 500 mM), resulting in final concentrations of 0.5, 1-, 5-, and 10-mM LC, respectively. On d6 and d8 of cooled storage, the semen samples were assessed as described.

#### Sperm motility and kinematic analysis

Following the addition of various LC concentrations to the cooled semen samples, sperm motility and kinematic parameters were assessed using a computer-assisted sperm cell analysis (CASA) system (Sperm Class Analyzer [SCA] 2005; Microptic). The following motility and kinetic parameters were measured using CASA: velocity curvilinear (VCL, μm/s), velocity straight line (VSL, μm/s), velocity average path (VAP, μm/s), linearity (LIN, %), straightness (STR, %), wobble (WOB, %), amplitude of lateral head displacement (ALH, μm), beat– cross frequency (BCF, Hz) and percentage of total (non-static) sperm motility (TM). A 5 μL semen sample was placed on a slide, covered with a coverslip (22 × 22 mm) and observed with a phase contrast microscope (×100) linked to the CASA. A total of nine fields per sample were analysed. The CASA settings used were: The CASA set-up was captured: 25 images per second; optics: Ph-; particle area greater than 10 and smaller than 80 μm^2^; VCL slow: between 10 and 25, medium: between 25 and 45, and rapid: greater than 45 μm/s; progressive motility: greater than 45% of STR and circular motility: smaller than 50% LIN.

#### Assessment of sperm membrane functionality

To evaluate sperm membrane functionality, a hypoosmotic swelling test (HOST) was conducted. Semen was added to distilled water at a ratio of 1:2 (Lagares et al., 2000), incubated in a water bath (37 °C, 5 min) and observed with phase-contrast microscopy (x400) (Lagares et al., 1998). To calculate the percentage of HOST+ sperm with swollen tails, the number of sperm with bent tails identified during the morphological evaluation was subtracted.

#### Concentration of nitrite and hydrogen peroxide

To assess the antioxidant effect of LC and the control group, the concentrations of NO_2_^−^ and H_2_O_2_ (μM/μg protein) in the cooled semen were analyzed. As NO_2_^−^ is one of the two primary, stable, and non-volatile breakdown products of nitric oxide (NO), it was quantified using spectrophotometry with Griess reagent (Green et al., 1982). This method helps measure NO formation within the sperm. The Griess reagent system relies on a chemical diazotization reaction involving 2% (w/v) sulphanilamide and 0.2% (w/v) N-(1-naphthyl) ethylenediamine dihydrochloride (NED) under acidic conditions (5% [v/v] phosphoric acid). The limit of detection was 2.5 μM (125 pmol) NO_2_^−^ (in ultrapure, deionized distilled water). The samples’ absorbance was measured at 540 nm. Hydrogen peroxide concentrations were measured using the FOX2 modified method (Nouroozzadeh et al., 1994). This technique involves the oxidation of ferrous ions (Fe^2+^) to ferric (Fe^3+^) under acidic conditions by lipid hydroperoxides. The indicator used was xylene orange, which reacts with Fe^3+^ ions producing a blue-purplish chromophore with a coefficient of extinction of 4.3 × 10^4^ M^−1^ cm^−1^ at 560 nm. H_2_O_2_ concentrations were determined based on the molar extinction coefficient of hydroperoxide using the following equation:


A_ = ε_. C (1)

where Aλ = absorbance at 560 nm; Ԑλ = molar extinction coefficient of the chromophore; C = hydroperoxide concentration (mol/mL).

### Experiment 2: Assessment of sperm ZP binding with LC addition

On the d6 of cooled storage, tubes containing a pool of 10 cooled boar insemination doses were placed at 30° and incubated for 90 minutes at 37 °C to the swim-up selection. Sperm were aspirated from the surface of each tube and the concentration/ mL was calculated with a hemocytometer. The medium used was TCM 199 with Hank’s salts supplemented with 10% bovine fetal serum (BFS), 0.1mg/mL streptomycin, and 100 IU /mL penicillin. The semen samples were extended to a final concentration 0.5x10^6^ sperm per doses, and two treatments were performed: one with extended semen (control) and another containing the LC concentration that yielded the best results in Experiment 1.

Porcine oocytes were obtained from ovaries of sows slaughtered in an abattoir. Each group of 30 oocytes was incubated at 38.5 °C in a 5% CO_2_^-^ atmosphere in 200µL droplet of TCM 199 medium with Hank’s salts + 10% BFS + 0.1mg/mL streptomycin + 100UI/mL penicillin until the zona binding assay was conducted. Each droplet containing 30 oocytes was inseminated with a final concentration of 0.5x10^6^ sperm stained with 10 μL of Hoechst 33342 solution (10 mg of Hoechst per 1 mL of distilled water), for each treatment and co-incubated at 38.5 °C with 5% CO_2_^-^ atmosphere for 20 minutes. The sperm and oocytes of each treatment were observed using an epifluorescent microscope (filter ex, 361 nm; em, 486 nm) und the number of sperm- ZP binding were calculated. (Martins et al., 2018).

### Experiment 3: Addition of LC to semen after one day of cooled storage

After one day of storage (d1) at 17 °C, 10 porcine insemination doses (containing 100 mL each) were distributed in two 10 mL samples. One sample served as the control (without LC), and the other contained the LC concentration that yielded the best results in Experiment 1. These samples were assessed on days 5, 7, 9, and 12 of cooled storage, following the same procedures described in Experiment 1, including assessment of sperm membrane integrity.

#### Sperm membrane integrity evaluation

Sperm membrane integrity assessment was conducted using epifluorescence microscopy, employing Hoechst 33342 (filter ex, 361 nm; em, 486 nm) and propidium iodide (PI; filter ex, 535 nm; em, 617 nm) probes. A total of 10 μL of Hoechst solution (10 mg of Hoechst per 1 mL of distilled water), 10 μL of PI solution (0.5 mg of PI per 1 mL of 0.9% saline solution), and 10 μL of formal citrate solution (1.7 mM) were added to a 100 μL semen sample. After incubation for 10 minutes at 37 °C, a droplet of the stained sample was placed on a microscope slide and covered with a coverslip. The stained sample was then examined using epifluorescence microscopy (× 1000). Sperm with red-stained heads (PI-positive) were counted as having damaged membranes (nonviable), while all sperm with blue staining (Hoechst-positive and PI-negative) were counted as having undamaged membranes (viable) and a total of two hundred spermatozoa were evaluated for each sample.

### Statistical analysis

An experimental design with randomized blocks, using a porcine dose as a block, was used. The mean values of sperm analysis were analyzed using Analysis of Variance (ANOVA) and compared with the Duncan test and Kruskal Wallis. The statistical analysis of sperm ZP binding was performed using ANOVA, and the mean values were compared with the Kruskal-Wallis and Dunn tests. All data were analyzed using the Infostat program (FCA, Universidad Nacional de Córdoba, Argentina) and a probability of *P* < 0.05 was considered significant. When no interactions among time and treatment evaluated were observed, treatments’ mean values were presented.

## Results

### Experiment 1

CASA endpoints (mean ± SEM) were assessed after the addition of LC to cooled porcine semen on day 5, with evaluations conducted on days 6 and 8, as shown in Table 1. Parameters including LIN, STR, ALH, and BCF showed no significant differences among experimental groups during that period (*P*>0.05). Additionally, sperm TM, PM and kinematic parameters rapid, VCL, and VAP decreased significantly from day 6 to day 8 of cooling (*P*<0.05). On the other hand, on day 8, the percentage of sperm with TM in the 10 mM LC treatment was higher (52.2% ± 5.2%) compared to that in the control group (28.7% ± 6.3%, P<0.05).

**Table 1:** CASA end points (mean ± SEM) after adding LC to cooled porcine semen on day 5 and evaluating on days 6 and 8.

	**LC (mM)**	**Day 6**	**Day 8**
TM	0	71.5 ± 8.2^a^	28.7 ± 6.3 ^c^
(%)	0.5	74.9 ± 3.7^a^	42.5 ± 7.7 ^bc^
	1	81.5 ± 3.8^a^	45.6 ± 6.9 ^bc^
	5	78.9 ± 4.4^a^	42.4 ± 6.5 ^bc^
	10	72.9 ± 3.9 ^a^	52.2 ± 5.2 ^b^
PM	0	41.0 ± 7.6^a^	12.5 ± 4.6 ^b^
(%)	0.5	37.4 ± 3.6 ^a^	15.7 ± 4.2 ^b^
	1	46.8 ± 5.2 ^a^	12.3 ± 3.4 ^b^
	5	43.9 ± 6.4 ^a^	15.4 ± 5.3 ^b^
	10	36.2 ± 4.7 ^a^	20.3± 4.2 ^b^
Rapid	0	36.3 ± 6.5 ^a^	10.9 ± 3.6 ^b^
(%)	0.5	44.0 ± 4.9 ^a^	15.1 ± 4.3 ^b^
	1	45.6 ± 4.8 ^a^	16.3 ± 5.6 ^b^
	5	44.6 ± 5.3 ^a^	11.2± 2.1 ^b^
	10	39.4 ± 4.5 ^a^	15.1 ± 2.9 ^b^
VCL	0	48.0 ±3.3 ^a^	35.2 ± 3.3 ^b^
(µm/s)	0.5	56.6 ± 3.8^a^	36.1 ± 2.9 ^b^
	1	53.2 ± 3.2 ^a^	38.0 ± 3.7 ^b^
	5	53.1 ± 2.8 ^a^	35.0 ± 2.5 ^b^
	10	52.0 ± 2.8 ^a^	35.3 ± 2.4 ^a^
VSL	0	19.8 ± 2.2 ^abc^	14.0 ± 2.4 ^cd^
(µm/s)	0.5	19.8 ± 1.2 ^abc^	12.5 ± 1.1 ^d^
	1	21.3 ± 1.9 ^ab^	16.0 ± 2.8 ^abcd^
	5	21.5 ± 2.1 ^a^	12.0 ± 1.2 ^d^
	10	20.1 ± 1.7 ^ab^	15.4 ± 1.4 ^bcd^
VAP	0	32.2 ± 2.5 ^ab^	21.8 ± 2.9 ^c^
(µm/s)	0.5	35.5 ± 2.5 ^a^	21.6 ± 2.0 ^c^
	1	35.0 ± 2.5 ^a^	25.4 ± 3.4 ^bc^
	5	35.9 ± 2.6 ^a^	20.1 ± 1.4 ^c^
	10	35.0 ± 2.7 ^a^	23.6 ± 1.9 ^c^
LIN	0	41.6 ± 4.4	38.9 ± 3.8
(%)	0.5	35.8 ± 1.8	35.3 ± 2.2
	1	40.3 ±3.3	42.1 ± 5.24
	5	40.2 ± 3.3	35.3 ± 3.6
	10	38.8 ± 3.0	43.9 ± 2.8
STR	0	60.7±3.9	62.9 ± 3.1
(%)	0.5	57.0 ± 2.7	58.6 ± 2.3
	1	60.6 ± 2.8	61.5 ± 3.8
	5	59.3 ± 3.1	59.3 ± 3.2
	10	57.8 ± 3.4	65.3 ± 2.6
ALH	0	2.2 ± 0.1 ^ab^	2.0 ± 0.2 ^b^
(µm)	0.5	2.5 ± 0.1 ^a^	2.0 ± 0.3 ^ab^
	1	2.4 ± 0.1 ^ab^	2.3 ± 0.2 ^ab^
	5	2.3 ± 0.1 ^ab^	2.4 ± 0.1 ^ab^
	10	2.3 ± 0.1 ^ab^	2.1 ± 0.1 ^ab^
BCF	0	6.9 ± 0.5 ^ab^	6.2 ± 0.9 ^ab^
(Hz)	0.5	7.5± 0.3 ^ab^	5.9 ± 0.7 ^b^
	1	7.4± 0.3 ^ab^	6.6 ± 0.3 ^ab^
	5	7.1 ± 0.1 ^ab^	7.7 ± 0.4 ^a^
	10	7.3 ± 0.2 ^ab^	6.8 ± 0.6 ^ab^

^abcd^ Values with a different superscript differed (P <0.05). TM: total motility; PM: progressive motility; VCL: curvilinear speed; VSL: straight line; VAP: average path velocities; LIN: linearity; STR: straightness; ALH: amplitude of lateral head displacement; BCF: beat cross frequency; LC 0: control; LC 0.5-10: 0.5-10mM l-carnitine.

The percentage of functional sperm membrane (HOST+) remained consistent from day 6 to day 8 of cooling (*P* > 0.05, Table 2). However, the mean value over both evaluated days was higher in treatments with higher LC concentrations (1-, 5-, and 10-mM LC) compared to the control (Table 2, *P* < 0.05)

**Table 2:** Percentage of functional sperm membrane (HOST+) after adding LC to cooled porcine semen on day 5 and evaluating on days 6 and 8 (Mean ± SEM).

	**LC (mM)**	**Day 6**	**Day 8**	**Mean ± SEM**
HOST+	0	37.8 ± 4.3 ^abcd^	30.7 ± 2.8 ^d^	34.3 ± 2.7 ^b^
(%)	0.5	44.0 ± 2.9 ^abc^	35.0 ± 2.7 ^cd^	39.5 ± 2.2 ^ab^
	1	44.0 ± 4.9 ^abc^	40.9 ± 2.6 ^abcd^	42.5 ± 2.8 ^a^
	5	48.3 ± 4.7 ^ab^	37.5 ± 2.8 ^bcd^	42.9 ± 3.0 ^a^
	10	49.4 ± 4.6 ^a^	38.2 ± 2.4 ^abcd^	43.8 ± 2.8 ^a^

^ab^ Values with a different superscript differed (*P* < 0.05). LC 0: control; LC 0.5-10: 0.5-10mM l-carnitine.

Hydrogen peroxide and nitrite concentrations remained constant from day 6 to day 8 of cooling and showed no significant differences among experimental groups on the evaluated days (Table 3, *P* > 0.05).

**Table 3:** Concentrations of hydrogen peroxide (H_2_O_2_) and nitrite (NO_2_^-^, μM/μg de protein) after adding LC to cooled porcine semen on day 5 and evaluating on days 6 and 8 (Mean ± SEM).

	**LC (mM)**	**Day 6**	**Day 8**	**Mean ± SEM**
H_2_O_2_	0	60.9 ± 8.6 ^ab^	72.0 ± 6.5 ^a^	65.8 ± 5.5
(μM/μg protein)	0.5	55.3 ± 3.8 ^ab^	63.2 ± 8.3 ^ab^	59.3 ± 4.5
	1	57.3 ± 5.9 ^ab^	53.7 ± 5.6 ^ab^	55.5 ± 4.0
	5	59.6 ± 8.9 ^ab^	59.1 ± 6.9 ^ab^	59.7± 5.5
	10	44.2 ± 4.2 ^b^	61.4 ± 7.6 ^ab^	52.8± 4.7
NO_2_^-^	0	35.2 ±5.2	34.0 ± 5.2	34.6 ± 3.6
(μM/μg protein)	0.5	31.8 ±2.3	39.9 ± 9.5	35.8 ± 4.8
	1	33.0 ±3.6	29.4 ± 4.1	31.2 ± 2.7
	5	34.4 ±5.5	30.2 ± 4.6	32.3 ± 3.5
	10	26.8 ± 2.9	31.3 ± 6.3	29.0 ± 3.4

^ab^ Values with a different superscript differed (*P* < 0.05). LC 0: control; LC 0.5-10: 0.5-10mM l-carnitine.

### Experiment 2

The concentration of LC selected for testing in the third experiment was 10 mM. This choice was based on the results of the first experiment, where the addition of 10 mM LC to cooled semen led in increased percentages of total motility (TM) compared to the control group. Consequently, in the second experiment, our objective was to ascertain whether the addition of 10 mM LC on day 6 of storage increased the number of sperm bound to the zona pellucida (ZP) compared to the control group. The addition of 10 mM LC to cooled semen at d6 of storage increased the number of sperm bound to ZP compared to the control (27.7 ± 1.4 *vs*. 14.2 ± 1.1 sperm, P<0.05).

### Experiment 3

The concentration of LC selected for testing in the third experiment was 10 mM. This choice was based on the results of the first experiment, where the addition of 10 mM LC to cooled semen on day 5 (d5) resulted in increased percentages of total motility (TM) and functional sperm membrane on day 8 (d8) compared to the control group. Therefore, in the third experiment, we aimed to determine whether the addition of 10 mM LC to cooled porcine semen after the first day of semen storage would lead to improvements and sustained semen characteristics. The analysis was performed on days 5, 7, 9, and 12 to track any potential long-term effects of LC supplementation on semen quality.

No significant differences were observed among the experimental groups on days 5, 7, and 9 of semen storage (Table 4). However, on day 12, the addition of LC resulted in significant improvements compared to the control group. Specifically, LC increased the following sperm characteristics: TM (88.8 *vs* 64.0%), rapid (43.5 *vs* 22.5), VAP (30.6 *vs* 22.9 µm/s), VCL (48.6 *vs* 34.4 µm/s), ALH (2.4 *vs* 2.0 µm) and BCF (2.5 *vs* 2.0 Hz) compared to the control (Table 4, *P* < 0.05).

**Table 4:** CASA endpoints, presented as both mean ± SEM and median (minimum - maximum), assessed after adding LC to cooled porcine semen following one day of storage and evaluating on days 5, 7, 8, and 12.

	**LC**	**Day 5**	**Day 7**	**Day 9**	**Day 12**
TM	0	81.4 (70.0 - 90.7) ^ab^	79.1 (49.2- 92.2) ^ab^	81.6 (18.9 - 91.3) ^ab^	64.0 (39.5 - 95.2) ^a^
(%)	10	91.1 (33.6 - 96.8) ^b^	82.0 (66.8 - 91.2) ^ab^	90.8 (45.50- 99.3) ^b^	88.8 (47.9 - 99.8) ^b^
PM	0	44.8 ± 4.0	45.7 ± 4.4	35.2 ± 6.1	31.4 ± 6.4
(%)	10	44.7 ± 6.9	44.9 ± 4.0	46.2 ± 7.6	49.1 ±6.0
Rapid	0	43.3 ± 5.0 ^a^	33.0 ± 3.9 ^ab^	28.3 ± 6.0 ^ab^	22.5 ± 6.0^b^
(%)	10	43.7 ± 7.0 ^a^	36.2 ± 3.2 ^ab^	41.8 ± 8.2 ^ab^	43.5 ± 9.0^a^
STR	0	63.2 ± 2.5	67.7 ± 1.8	64.6 ± 1.6	69.2 ± 1.5
(%)	10	64.6 ± 2.6	65.9 ± 2.5	64.0 ± 1.0	67.3 ± 1.4
LIN	0	43.5 ± 2.7 ^ab^	47.7 ± 2.3 ^a^	41.7 ± 1.9 ^ab^	45.3 ± 2.6 ^ab^
(%)	10	44.2 ± 3.3 ^ab^	48.5 ± 2.5 ^a^	39.9 ± 1.6 ^b^	42.9 ± 1.9 ^ab^
VAP	0	35.2 ± 2.6 ^a^	31.4 ± 2.1 ^a^	26.2 ± 2.2 ^ab^	22.9 ± 3.0 ^b^
(µm/s)	10	33.1 ± 2.8 ^a^	34.3 ± 2.3 ^a^	29.4 ± 4.0 ^ab^	30.6 ± 3.0 ^a^
VCL	0	51.1 ± 2.9 ^a^	44.6 ± 2.6 ^a^	40.6 ± 3.2 ^ab^	34.4 ± 3.4 ^b^
(µm/s)	10	49.5 ± 4.4 ^a^	46.7 ± 3.1 ^a^	46.6 ± 5.4 ^a^	48.6 ± 5.2 ^a^
VSL	0	22.2 ± 1.8 ^a^	21.2 ± 1.4 ^ab^	17.2 ± 1.8 ^ab^	16.0 ± 2.4 ^b^
(µm/s)	10	21.0 ± 1.6 ^ab^	22.3 ± 1.3 ^a^	18.9 ± 2.6 ^ab^	20.4 ± 1.8 ^ab^
ALH	0	2.3 (2.1 - 2.7) ^bcd^	2.2 (1.7 -2.5) ^abc^	2.4 (1.5 - 2.8) ^bcd^	2.0 (1.3 – 2.5) ^a^
(µm)	10	2.3 (1.5 - 3.3) ^bcd^	2.1 (1.9 – 2.7) ^ab^	2.5 (2.3 – 3.4) ^d^	2.4 (1.9 -3.4) ^cd^
BCF	0	2.4 ± 0.3 ^a^	2.2 ± 0.3 ^a^	2.4 ± 0.3 ^a^	2.0 ± 0.6 ^b^
(Hz)	10	2.4 ± 0.3 ^a^	2.1 ± 0.2 ^a^	2.6 ± 0.3 ^ab^	2.5 ± 0.3^a^

^abcd^ Values with different superscript differ (*P* < 0.05). TM: total motility; PM: progressive motility; STR: straightness; LIN: linearity; VAP: average path velocities; VCL: curvilinear speed; VSL: straight line; ALH: amplitude of lateral head displacement; BCF: beat cross frequency; LC 0: control; LC 10: 10 mM l-carnitine.

The percentage of sperm with a functional plasma membrane decreased only on day 12 of cooling in both treatments compared to that on day 9 (Table 5, *P* > 0.05). Sperm membrane functionality and integrity did not show any significant differences between the treatments during the 12 days of analysis (*P* > 0.05, Table 5).

**Table 5:** Percentage of functional (HOST+) and intact sperm membrane of cooled porcine sperm assessed after adding LC to semen following one day of storage and evaluated on days 5, 7, 8 and 12 (Mean ± SEM and median (minimum-maximum)).

	**LC**	**Day 5**	**Day 7**	**Day 9**	**Day 12**
HOST+	0	49.5 ± 3.49 ^a^	51.4 ± 4.07 ^a^	57.5 ± 5.08 ^a^	31.9 ± 4.85 ^b^
(%)	10	50.2 ± 3.94 ^a^	57.2 ± 3.57 ^a^	53.9 ± 4.33 ^a^	31.3 ± 6.33 ^b^
Integrity	0	89.6 (58.7 - 99.2) ^ab^	92.2 (65.8 - 95.6) ^b^	87.4 (55.7 - 98.5) ^a^	83.8 (11.9 - 91.3) ^a^
(%)	10	85.2 (59.0 - 98.5) ^ab^	92.3 (72.8 - 97.6) ^b^	90.4 (32.5 - 93.2) ^ab^	81.7 (46.0 - 92.2) ^a^

^ab^ Values with different superscript differ (*P* < 0.05), Integrity: percentage of sperm with intact membrane. LC 0: control; LC 10: 10 mM l-carnitine.

**Figure 2:**
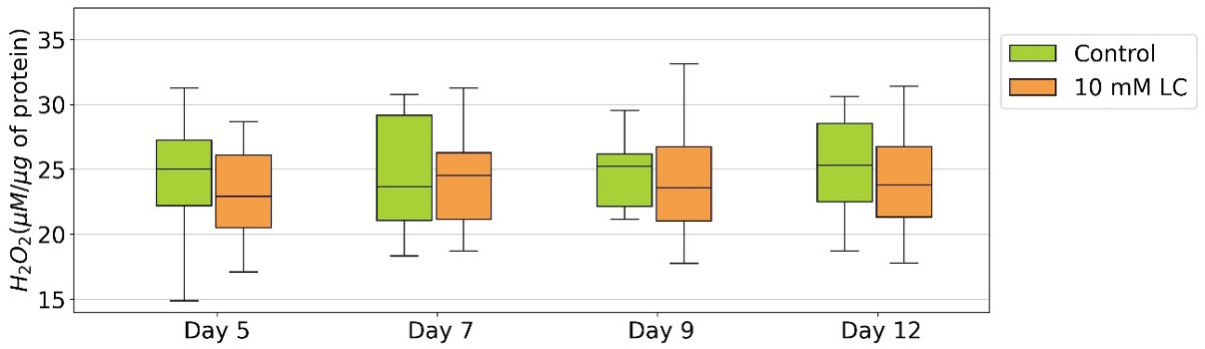
Hydrogen peroxide (H_2_O_2_) concentration (μM/μg protein) of cooled porcine sperm assessed after adding LC to semen following one day of storage and evaluated on days 5, 7, 8 and 12.

**Figure 3:**
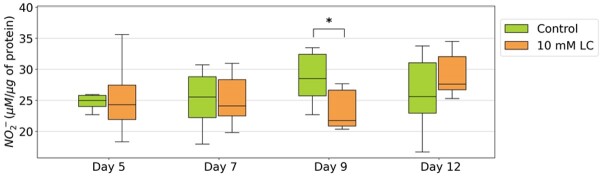
Nitrite (NO_2_^-^) concentration (μM/μg protein) of cooled porcine sperm assessed after adding LC to semen following one day of storage and evaluated on days 5, 7, 8 and 12. Values with * differ significantly (P < 0.05).

Hydrogen peroxide concentration did not exhibit significant differences among the various time points and treatments (Figure 2, *P* > 0.05). In the control treatment, NO_2_^-^ concentration remained constant until day 12 of cooled semen storage (Figure 3, *P* > 0.05). However, on day 9, LC supplementation led to a decrease in NO_2_^-^ concentration compared to the control (21.7% vs. 28.5%, P<0.05).

## Discussion

In the present study, the addition of 10 mM LC improved and prolonged sperm total motility (TM) for at least three days beyond the typically recommended usage of insemination doses (five days) and when it was added to semen on day 1 of storage, it extended sperm kinematic characteristics to day 12. These findings align with reports in other species demonstrating the positive effects of LC addition on sperm motility and kinematic parameters. LC supplementation improved sperm motility and kinematic characteristics in frozen epididymal cat sperm (25 mM, Manee-In et al., 2014), cooled equine semen stored for 48 hours (0.1 mM/mL, Lisboa et al., 2014), and post-thawed equine semen (1 mM, Lagares et al., 2022). These results are consistent with the increased number of sperm bound to ZP observed in the LC treatment during the present study. The interaction with the oocyte plasma membrane appears to explain much of the variability in sperm fertilizing potential among fertile boars and has been correlated with in vivo fertility (Berger et al., 1996). Therefore, in the present study, the enhanced sperm ZP- binding capability with LC addition seemed to be due to its positive effects on sperm metabolism.

Since porcine sperm motility typically declines after three days of storage (Storey, 1997; Awda et al., 2009), semen extenders are used to mitigate the detrimental effects of storage on sperm viability, thereby prolonging its longevity to at least five days (Gibb et al., 2015; Menegat et al., 2017). The sperm motility decrease during *in vitro* storage, might be a consequence of the increase in reactive oxygen species (ROS) as byproducts of sperm metabolism that disrupts sperm homeostasis and depletes energy (Gibb et al., 2015; Cerolini et al., 2000). In the present study, when LC was added to semen on day 1 of storage, it decreased NO_2_^-^ concentration on d9 exhibiting antioxidant properties. Nitric oxide (NO) is a free radical that plays a role in sperm physiology, motility, acrosome reaction and is produced by the intracellular enzyme nitric oxide synthase (NOS) (Staicu and Parra, 2017). In high concentrations, it increases ROS and RNS production leading to a decrease of sperm motility and membrane integrity (Balercia et al., 2005). Furthermore, since the sperm plasma membrane is rich in lipids, contributing to its fluidity, this also makes the membrane more susceptible to lipid peroxidation (Sanocka and Kurpisz, 2004). ROS attacks on sperm membranes disrupt their structure, altering fluidity and permeability (Flesch and Gadella, 2000).

Most studies assess the impact of oral l-carnitine supplementation on semen quality (Khaw et al., 2020; Nazari et al., 2021; Szymański et al., 2021; Balogun et al., 2022; Ma and Sun, 2022; Chang et al., 2023; Lahimer et al., 2023). However, it is possible that when directly added to the semen, its action mechanism remains unchanged. LC facilitates the transfer of lipids and other substrates across the mitochondrial membrane for use in sperm energy production, reduces lipid availability for peroxidation and ROS formation, thereby stabilizing the membrane (Jeulin and Lewin, 1996; Gibb et al., 2015). *In vivo* studies have demonstrated that LC can reduce the concentration of malondialdehyde (MDA), a marker of lipid peroxidation, ensuring efficient fatty acid oxidation and preventing the accumulation of damaged lipids (Cabral et al., 2018). Elevated lipid peroxidation can have detrimental effects on the functional integrity and fluidity of the sperm plasma membrane due to its high levels of polyunsaturated fatty acids (PUFAs), rendering it particularly susceptible to damage (Hosen et al., 2015). As a result of lipid peroxidation, compounds such as MDA and 4- hydroxynonenal (4-HNE), further compromising sperm quality (Gomez et al., 1998). In a study, supplementation with LC was found to enhance seminal antioxidant activity, prevent MDA production, and protect sperm during storage in the epididymis and oviduct in aged roosters (Elokil et al., 2019). However, in the present study the direct access to lipid peroxidation through measurements of MDA and 4-HNE production was not performed. On the other hand, it is possible that LC acts indirectly decreasing lipid peroxidation by scavenging ROS and reducing oxidative stress, improving sperm parameters such as motility, morphology, and concentration (Mateus et al., 2023). In this study, when 1-, 5-, and 10-mM LC were added to semen on day 5 of storage, sperm membrane functionality increased. This may have resulted from a decrease in ROS availability, leading to enhanced membrane stabilization (Gibb et al., 2015). Similarly, in this study, when LC was added at the beginning of storage, it acts as an antioxidant reducing NO_2_^-^ concentration. Thus, the action of LC could potentially mitigate lipid peroxidation, suggesting a beneficial effect on boar sperm quality.

The LC solution can be stored by freezing at -20 °C. Prior to adding the LC solution to the semen doses, it should be thawed and then mixed with boar semen at a temperature of 16 °C, which is consistent with the temperature of cooled stored boar semen. This process ensures that the quality of the cooled semen will not be compromised until it is ready for use. This procedure could be a valuable practice for the suine industry, especially crucial for boars with sperm that do not tolerate longer storage periods, ensuring a high-quality cooled semen for up to 12 days. Thus, in addition to its positive effects on sperm metabolism and ZP- binding, LC also appears to have antioxidant properties and protects sperm membrane functionality in porcine sperm. These findings suggest that the addition of 10 mM LC to cooled porcine semen may potentially enhance the fertility of porcine semen doses up to 12 days.

## Conclusion

The addition of LC both at days 1 and 5 of storage to cooled porcine semen seemed to be advantageous in extending the viability of porcine sperm doses. The improvements in sperm characteristics and the increased number of sperm bound to ZP after five day of storage suggest that adding 10 mM LC to porcine semen doses might potentially enhance the fertility of porcine semen. Therefore, the addition of LC to cooled semen could be a valuable practice for the suine industry, ensuring a high-quality cooled semen for up to 12 days. This could be especially crucial for boars with sperm that do not tolerate longer storage periods.
